# One-carbon metabolism is distinct metabolic signature for proliferative intermediate exhausted T cells of ICB-resistant cancer patients

**DOI:** 10.1038/s41420-025-02332-z

**Published:** 2025-02-14

**Authors:** Ye-Chan Park, Yeseong Hwang, Jae Woong Jeong, Chae Min Lee, Minki Kim, Sugyeong Jo, Seyeon Joo, Nahee Hwang, Sungsoon Fang

**Affiliations:** 1 Graduate School of Medical Science, Brain Korea 21 Project, Yonsei University College of Medicine, Seoul, 03772 Republic of Korea; 2 Department of Internal Medicine, Yonsei University College of Medicine, Seoul, 03722 Republic of Korea; 3 Department of Medicine, Yonsei University College of Medicine, Seoul, 03722 Republic of Korea; 4 Department of Biomedical Sciences, Gangnam Severance Hospital, Yonsei University College of Medicine, Seoul, 06273 Republic of Korea

**Keywords:** Cancer microenvironment, Cancer metabolism, Tumour immunology

## Abstract

One-carbon metabolism (1CM) has been reported to promote cancer progression across various malignancies. While 1CM is critical for cell proliferation by enhancing nucleotide synthesis, its physiological roles within different cell types in the tumor immune microenvironment (TIME) still remain unclear. In this study, we analyzed bulk-RNA sequencing and single-cell RNA sequencing (scRNA-seq) data from lung adenocarcinoma (LUAD) patients to elucidate the functional roles of 1CM within the TIME. Moreover, we examined scRNA-seq data from patients treated with immunotherapy across various cancers, including LUAD, glioblastoma, renal cell carcinoma, colorectal cancer, and triple-negative breast cancer. Compared to other cell types, 1CM gene profiles are significantly enriched in a specific subset of T cells. Intriguingly, these high-1CM T cells are identified as proliferative intermediate exhausted T cells (Tex^int^). Furthermore, these proliferative Tex^int^ received the most robust CD137 signaling. Consistently, analysis of scRNA-seq data from LUAD patients undergoing anti-PD1 immunotherapy demonstrated that proliferative Tex^int^ exhibited higher 1CM scores and increased CD137 signaling. This observation was particularly pronounced in non-responders to immunotherapy, where the Tex^int^ population was significantly expanded. We further established that 1CM is a prominent signaling pathway in proliferative Tex^int^ in patients resistant to immunotherapy across multiple cancer types. Collectively, we identify CD137 signaling as a distinctive pathway in proliferative Tex^int^ of LUAD patients who do not respond to immunotherapy. These findings propose that targeting 1CM may represent a novel therapeutic strategy to enhance the efficacy of immunotherapy by mitigating Tex^int^ proliferation in diverse cancers.

## Introduction

Despite constant decline in incidence, lung cancer is one of the leading cancer types associated with cancer-related death and estimated new cases [1, 2]. Non-small cell lung cancer (NSCLC) is a major type of lung cancer which contributes to about 85% of lung cancer [3]. Lung adenocarcinoma (LUAD) is predominant histological phenotype of NSCLC, evolves from epithelial cells located in mucosal glands, and represents about 50% of NSCLC and 40% of all lung cancers [4, 5]. LUAD is highly associated with previous smoking history, however it is also the most common subtype diagnosed in people never smoked [6].

One-carbon metabolism (1CM) is a series of metabolic processes originated from serine, followed by folate cycle and methionine cycle in both cytoplasm and mitochondria [7]. Serine, the initiating amino acid of 1CM, can be directly transported into cells by several transporters such as ASCT1, ASCT2, SNAT1, SNAT2, and ASC1 [8], and synthesized by de novo serine biosynthesis process from 3-phosphoglycerate (3-PG) which are generated in glycolysis [9]. Side products of 1CM can enhance cell proliferation by supporting various building blocks used in multiple physiological processes, including purine and thymidine synthesis, glutathione synthesis, amino acids metabolism (glycine, serine, and methionine), epigenetic regulation [10]. For these advantages, 1CM is favored by cancer cells. However, the expression and roles of 1CM-related genes in tumor microenvironment (TME), especially in tumor immunity, are still largely unknown.

Tumor immunity is characterized by immunosuppressive TME which makes it possible for cancer cells to evade immune surveillance. One of the well-known factors to lead tumor immune escape is increased expression of immune checkpoint molecules mediating co-inhibitory signals such as PD-1, CTLA-4, LAG3, TIM-3 in T cells, so-called T cell exhaustion [11]. Recently, immune checkpoint blockades (ICBs) such as pembrolizumab (anti-PD-1), and ipilimumab (anti-CTLA-4) are largely used in immunotherapy, showing almost complete tumor eradication in responders [12]. However, the majority of patients who received ICBs remained non-responding to treatment, while only 20–40% of patients responded [13]. Despite numerous efforts to enhance immunotherapy response in non-responders, the exact mechanism by which they do not respond to the treatment stays unclear.

To overcome these challenges in immunotherapy, there have been efforts to understand the process of T cell exhaustion at the molecular level. In many studies, the states of exhausted T cells are classified by their molecular phenotype; precursors of exhausted T cells (Tpex), intermediate exhausted T cells (Tex^int^), and terminal exhausted T cells (Tex^term^) [14–17]. Tpex are characterized by high expression of memory and stemness signatures such as TCF-1 (encoded by *TCF7*), CXCR5, Ly108 (encoded by *SLAMF6*), CD73 (encoded by *NT5E*), and ID3 [16–18]. Among them, TCF-1 is an essential transcription factor for maintaining their function as precursor [18]. Tpex can be activated to produce effective cytokines and kill target cells by interacting with dendritic cells (DCs) located in tumor-draining lymph nodes (TDLNs) or tertiary lymphoid structures [16, 19–25]. By contrast, downregulation of TCF-1 and upregulated TOX expression leads to T cell terminal exhaustion, generating Tex^term^ which are epigenetically scarred and almost lose their capacity to produce cytokines [16, 26–33]. Tex^int^ are in an intermediate stage of exhaustion, which are further differentiated from Tpex but not fully exhausted [16–18]. These Tex^int^, like Tpex, still exhibit limited cytokine production and cytotoxicity, which can be activated through interaction with DCs in TDLNs, potentially enhancing immunotherapy response [34–38]. However, Tex^int^ ultimately lose their effector functions and are converted into Tex^term^, contributing to immune suppression [16, 18, 37, 39].

CD137, encoded by *TNFRSF9* gene, is a TNFR superfamily member that has been known as a co-stimulatory receptor of T cells [40]. CD137 expression requires canonical TCR (T cell receptor) signals and B7 family-mediated CD28 co-stimulatory signals [41, 42]. Amplified CD137 molecules are loaded on surface of T cell membrane, binding with CD137 ligands (CD137L) and promote T cell proliferation, anti-apoptosis, cytokine secretion, chromatin remodeling, and mitochondrial fitness [43], inducing tumor regression in various mouse models [44–46]. However, in some studies, anti-CD137 agonists suppress immune activation in some autoimmune disease mouse models [47–50]. In addition, it is revealed TCR-independent CD137 signaling induces proliferation and terminal differentiation of CD8+ exhausted T cells [51], supporting double-edged roles of CD137 signaling, immune activation or suppression.

In this study, using public bulk and single-cell transcriptomics data, we discovered that Tex^int^ have high 1CM expression, especially mitochondrial folate cycle, and are significantly affected by CD137 signaling in LUAD patients. In addition, we found that these 1CM-enriched Tex^int^ are enriched in tumor samples and ICB-treatment non-responders than normal samples and responders, respectively.

## Results

### 1CM is highly upregulated leading to inferior outcome in LUAD patients

**Fig. 1:**
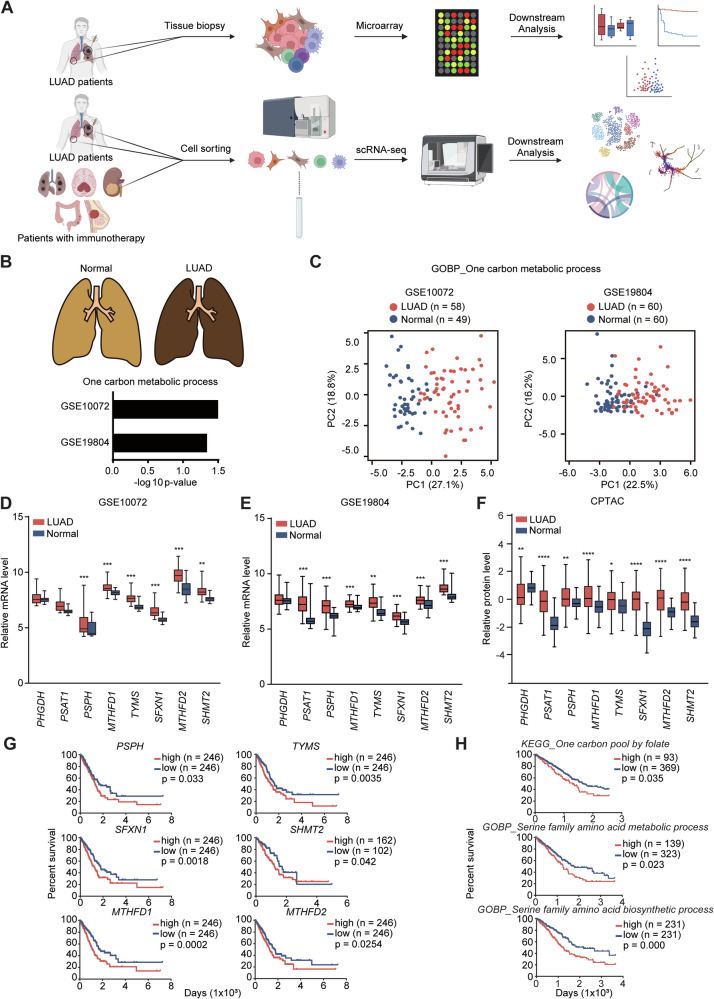
1CM is highly upregulated leading to poor prognosis in LUAD patients. **A** Total schematic diagram for analysis. Two bulk microarray and scRNA-seq data from LUAD patients and adjacent normal tissue were used in analysis, GSE10072 (LUAD; *n* = 58, Normal; *n* = 49), GSE19804 (LUAD; *n* = 60, Normal; *n* = 60), and GSE123904 (treatment-naïve LUAD; *n* = 7, adjacent normal tissue; *n* = 4). Additionally, five scRNA-seq data of immunotherapy responders and non-responders were employed to examine our findings in other cancer types, GSE207422 (LUAD, Pre_NR; *n* = 2, Post_NR; *n* = 4, Post_R; *n* = 2), GSE235676 (GBM, Post_R; *n* = 7, Post_NR; *n* = 5), EGAD00001008166 (ccRCC, Post_R; *n* = 1, Post_NR; *n* = 1), GSE205506 (CRC, Post_R; *n* = 5, Post_NR; *n* = 3), and GSE 169246 (TNBC, Pre_R; *n* = 5, Post_R; *n* = 2, Pre_NR; *n* = 4, Post_NR; *n* = 4). **B** GOBP analysis of DEGs shows one-carbon metabolic process was significant different between LUAD patients and normal subjects. **C** Two principal component analysis (PCA) show distinct expression pattern of one carbon metabolic process in LUAD and normal subjects. **D**, **E** Box plots of relative mRNA level expression associated with major 1CM-related genes show significant upregulation in LUAD patients compared to control in GSE10072 and GSE19804. Statistical significance was calculated using IDEP 1.1 webtool. **F** Box plot of 1CM-related protein expression reveals higher level in patients with LUAD through CPTAC dataset. Statistical significance was calculated by Wilcoxon rank-sum test. **G**, **H** Survival plots show poor prognosis in LUAD patients with high expression of 1CM-related genes and gene sets. The Log-rank (Mantel–Cox) test was used to calculate the *p* value.

The relationship between 1CM and LUAD was analyzed using two microarray datasets from LUAD tumor and adjacent normal lung tissue, accessible with GEO accession number GSE10074 and GSE19804, respectively. To examine the molecular signature of 1CM within tumor immune microenvironment (TIME) of LUAD patients, single-cell RNA-seq (scRNA-seq) data from treatment-naïve LUAD patients, accessible with GSE123904, was employed. For validation across different datasets, five scRNA-seq datasets of patients treated with immune checkpoint blockade (ICB) from various cancer types, including LUAD, glioblastoma (GBM), clear cell renal cell carcinoma (ccRCC), colorectal cancer (CRC), and triple negative breast cancer (TNBC) were analyzed. These datasets are available under GEO accession numbers GSE207422, GSE235676, EGAD00001008166, GSE169246, and GSE205506, respectively (Fig. 1A). Analysis of the two Gene Expression Omnibus (GEO) datasets revealed a distinct differential expression of one-caron metabolic processes between LUAD patients and normal controls (Fig. 1B). Principal component analysis (PCA) further demonstrated a clear segregation of one carbon metabolic genes between two groups (Fig. 1C). Subsequent examination of gene expression involved in 1CM indicated significantly elevated expression of genes related to de novo serine biosynthesis (*PHGDH*, *PSAT1*, *PSPH*) and folate cycle (*SFXN1*, *SHMT2*, *MTHFD1*, *MTHFD2*, *TYMS*) in LUAD patients, suggesting that LUAD tumors might have enriched 1CM compared to normal subjects (Fig. 1D–F). Survival analysis illustrated that patients with high expression scores of 1CM-related genes exhibited poor prognosis (Fig. 1G, H). Altogether, our bulk transcriptomics analysis demonstrated the potential biological roles of 1CM in the progression of LUAD, indicating its importance in the pathophysiology.

### Specific T cell cluster has higher enrichment of 1CM in LUAD patients

**Fig. 2:**
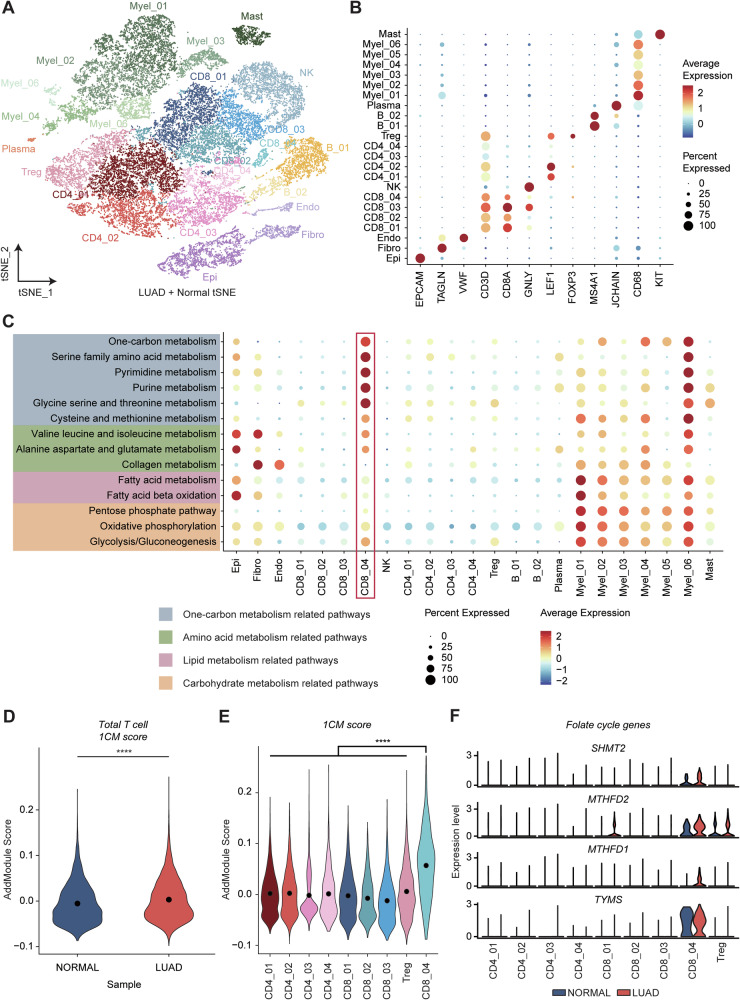
A specific T cell cluster exhibits higher enrichment of 1CM in LUAD patients. **A** t-SNE plot of scRNA-seq data represents total cell types identified within LUAD patients and their adjacent normal lung tissue samples. **B** Dot plot shows expression of marker genes in each cell type. **C** Dot plot portrays metabolic enrichment scores including 1CM-related pathways, amino acid metabolism-related pathways, lipid metabolism-related pathways, and carbohydrate-related pathways. Metabolic enrichment scores were calculated by AddModuleScore function in Seurat. **D** Violin plot shows 1CM score in overall T cell. 1CM score was calculated by AddModuleScore function in Seurat with GOBP one carbon metabolic process gene set. Statistical significance was calculated by Wilcoxon rank-sum test. **E** Violin plot shows different expression of 1CM in T cell subtypes. Statistical significance was calculated by Wilcoxon rank-sum test. **F** Violin plot reveals expression level of folate cycle-associated genes, *SHMT2*, *MTHFD2*, *MTHFD1*, and *TYMS*.

To investigate the relationship between 1CM and TIME in LUAD, we next analyzed scRNA-seq data from seven treatment-naïve LUAD samples and four normal lung samples. We characterized cell type heterogeneity using t-distributed Stochastic Neighbor Embedding (t-SNE) with gene markers, including epithelial cells (*EPCAM, MUC5AC*), fibroblasts (*TAGLN, ACTA2*), endothelial cells (*VWF, CLDN5*), CD8+ T cells (*CD3D, CD8A*), natural killer (NK) cells (*GNLY, NKG7*), CD4+ T cells (*CD3D, LEF1*), regulatory T cells (*CD3D, FOXP3*), B cells (*CD79A, MS4A1*), plasma cells (*CD79A, JCHAIN*), myeloid cells (*CD68, CD86*), and mast cells (*KIT, TPSAB1*) (Fig. 2A, B). Notably, T cells were present in highest proportions compared to other cells such as myeloid cells, B cells, and fibroblasts, underscoring the significance of T cells in the LUAD TIME (Fig. S1A). We observed differential preferences for metabolic processes among cell types using single-cell GSEA (ssGSEA) derived from Gene Ontology Biological Process (GOBP), Kyoto Encyclopedia of Genes and Genomes (KEGG), and HALLMARK gene sets (Fig. 2C). In addition, we analyzed ssGSEA results using all metabolism-related gene sets in KEGG (Fig. S1B). Epithelial cells were enriched for amino acid metabolism, including glutamate, arginine, phenylalanine, and tyrosine (Figs. 2C and S1B). Amino acid metabolism pathways involving collagen, tyrosine, and tryptophan were largely upregulated in fibroblasts, whereas myeloid cells were enriched with most of amino acid metabolism pathways, except for glutamate and glycosaminoglycan metabolism (Figs. 2C and S1B). Interestingly, T cells, particularly the CD8_04 subsets, exhibited a distinct preference for 1CM-related pathways (Figs. 2C and S1B). The 1CM score was significantly elevated in T cells from LUAD tissues compared to normal samples (Fig. 2D). Among T cell subsets, CD8_04 cells demonstrated a significantly higher mean expression and distribution of the 1CM score than other subtypes (Fig. 2E). Among various 1CM-related genes, several genes associated with the mitochondrial folate cycle, which is crucial for T cell proliferation [52, 53], were highly expressed in CD8_04 cells (Fig. 2F). These findings suggest that 1CM is enriched in the specific T cell subset CD8_04, particularly characterized by high expression of the mitochondrial folate cycle in LUAD patients.

### 1CM-enriched T cells are characterized as intermediate exhausted T cells (Tex^int^)

**Fig. 3:**
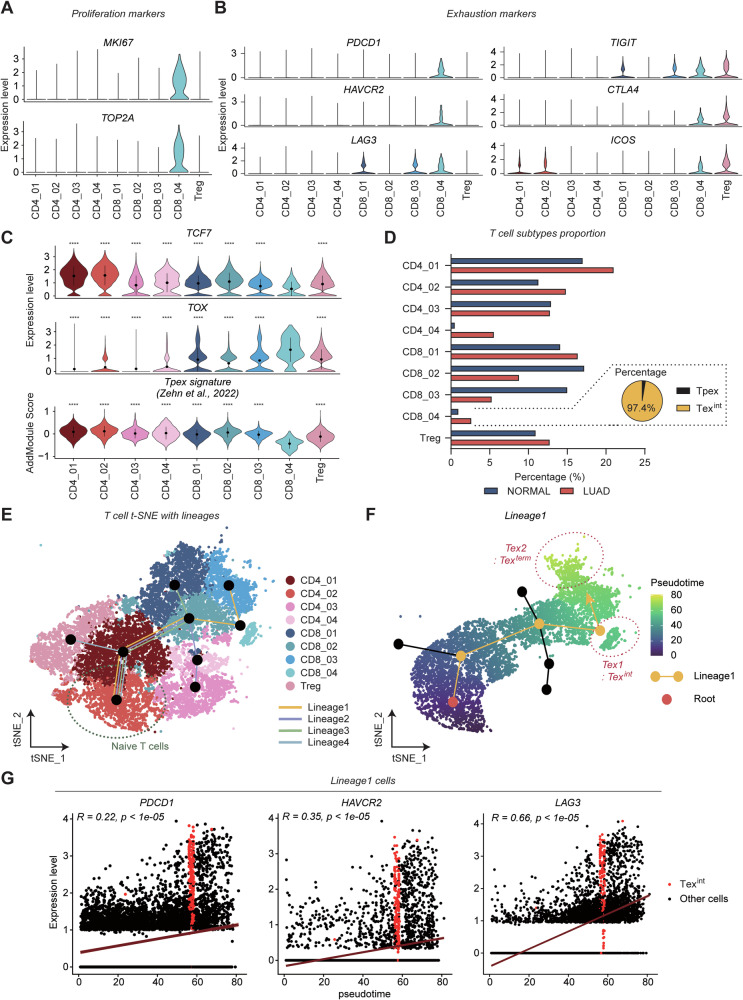
1CM-enriched T cells are characterized as Tex^int^. **A** Violin plot shows expression level of T cell proliferation markers, *MKI67* and *TOP2A* in T cell subtypes. **B** Violin plot represents expression level of several exhaustion markers such as *PDCD1*, *HAVCR2*, *LAG3*, *TIGIT*, *CTLA4*, and *ICOS* in T cell subtypes. **C** Violin plot depicts canonical Tpex marker *TCF7*, terminal exhaustion and differentiation marker *TOX*, and Tpex signature score. Tpex signature score was calculated by AddModuleScore function in seurat with five Tpex marker genes (*TCF7*, *CXCR5*, *SLAMF6*, *NT5E*, and *ID3*) derived from Zehn et al. [18]. Statistical significance was calculated by Wilcoxon rank-sum test. **D** Bar plot depicts the proportion of T cell subtypes. Particularly, the proportion of CD8_04 cells is higher in LUAD compared to normal. CD8_04 cells were divided into Tpex and Tex^int^ by Tpex signature score (Tpex; Tpex score >0, Tex^int^ ; Tpex score <0). The pie chart shows that Tex^int^ cells are dominant in CD8_04 cluster. **E** Slingshot exhibits the information of cluster and lineages in t-SNE plot of overall T cell clusters. Naïve T cells (CD4_02) are appointed as root cells. **F** Feature plot shows pseudotime distribution in lineage 1 containing Tex1 and Tex2, which is confirmed as Tex^int^ and Tex^term^, respectively. **G** Correlation plots show positive correlation of several exhaustion markers with pseudotime. Correlation was calculated as Pearson method. The *p* value was calculated by two-sided *t*-test. Tex^int^ cells were labeled as red color.

We next annotated each T cell subsets using specific marker genes to identify characteristics of 1CM-enriched T cells (CD8_04). The T cell subtypes were identified as Memory T cells (*TCF7*; CD4_01), Naïve T cells (*SELL*; CD4_02), Activated CD4 T cells (*PRKCE*, *TNF*; CD4_03, 04), Cytotoxic T cells (Tc cells) with distinct marker genes (*FASLG*; CD8_01, *GZMK*; CD8_02, *GZMB*; CD8_03), and regulatory T cells (*FOXP3*; Treg) (Fig. S2A). Notably, CD8_04 cells exhibited high expression levels of both proliferation markers (*MKI67 and TOP2A)*, and exhaustion markers (*PDCD1, HAVCR2*, *LAG3*, *TIGIT*, *CTLA4*, and *ICOS*) (Figs. S2A and 3A, B) [54, 55]. Based on the gene expression profiles of high proliferation capabilities and T cell exhaustion, we suspected that 1CM-enriched CD8_04 cells are precursors of exhausted T cells (Tpex) or intermediate exhausted T cells (Tex^int^), distinct with canonical terminal exhausted T cells (Tex^term^). To identify which signature is dominant in CD8_04 cluster between Tpex and Tex^int^, we checked canonical Tpex marker *TCF7* (encoding TCF-1), T cell terminal exhaustion marker *TOX*, and Tpex signature score (*TCF7, CXCR5, SLAMF6, NT5E, ID3*) (Fig. 3C). *TCF7* expression and Tpex signature score were decreased in CD8_04 cells while *TOX* expression was increased, suggesting that Tex^int^ are dominant in this cluster (Fig. 3C). Also, the proportion of CD8_04 cluster, containing 97.4% of Tex^int^, was increased in LUAD compared to normal samples (Fig. 3D).

To verify whether CD8_04 are Tex^int^, we assessed exhaustion signatures using ssGSEA with exhaustion markers, including *PDCD1* (encoding PD-1), *HAVCR2* (encoding TIM-3), and *LAG3*, and performed trajectory analysis. The ssGSEA results identified cells with dominant exhaustion signatures; Tex1 (originated from CD8_04), Tex2 (originated from CD8_03), and Tex3 (originated from CD8_01) (Fig. S3A). Also, expression levels of PDCD1, HAVCR2 and LAG3 were increased in Tex1, Tex2, Tex3 and some of Treg (Fig. S3A). The trajectory was generated using naïve T cells (CD4_02) as the root and revealed four distinct lineages (Fig. 3E). We next performed pseudotime analysis for each lineage (Figs. 3F and S3B). Given our hypothesis that Tex1 represents Tex^int^, we further focused on lineage1 which contains both Tex1 and Tex2 clusters. Intriguingly, pseudotime analysis indicated that Tex1 cells are differentiating into Tex2 cells (Fig. 3F). Correlation plots within lineage 1 also demonstrated a positive correlation between pseudotime and exhaustion markers (Figs. 3G and S3C). Proliferation markers were most highly expressed in Tex^int^, with their expression exponentially decreasing as pseudotime progressed (Fig. S3D). Collectively, these findings suggest that Tex1 can be regarded as 1CM-high Tex^int^, while Tex2 can be characterized as Tex^term^.

### CD137 signaling network is dominant incoming pathway to 1CM-enriched proliferative Tex^int^ in LUAD patients

**Fig. 4:**
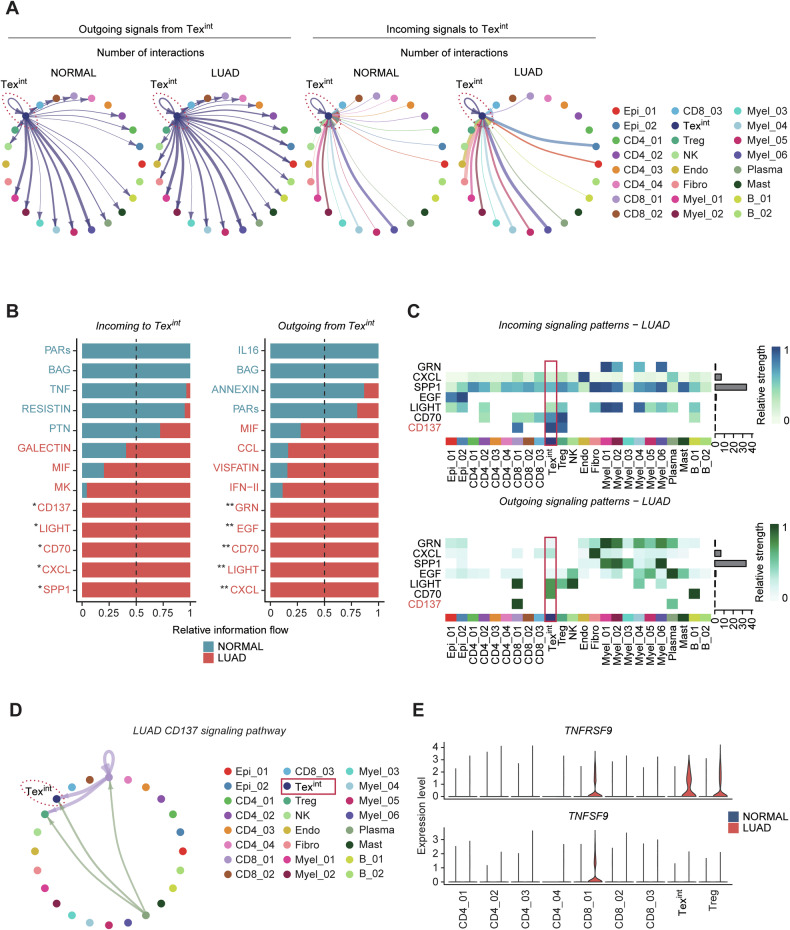
CD137 signaling network is a dominant incoming pathway to 1CM-enriched proliferative Tex^int^ in LUAD patients. **A** Circle plot shows number of interactions outgoing from Tex^int^ or incoming to Tex^int^ in total LUAD and normal clusters. Tex^int^ cluster was highlighted as a dotted line. **B** Bar plots exhibit difference in signal interactions between LUAD and normal Tex^int^. Exclusively observed signals in LUAD Tex^int^ were marked with asterisks (incoming signals; *, outgoing signals; **). **C** Heatmaps depict interaction patterns of outgoing signals and incoming signals in LUAD samples. Tex^int^ cluster was highlighted as a red box. **D** Circle plot shows Tex^int^ mainly receives CD137 signaling from plasma or CD8_01 cluster in LUAD samples. Tex^int^ cluster was highlighted as a dotted line. **E** Violin plot represents expression level of *TNFRSF9* (CD137) and *TNFSF9* (CD137L) in both LUAD and normal samples.

We next examined the potential cell-cell interactions of 1CM-enriched Tex^int^ in LUAD patients. With cell-cell communication analysis, we aimed to delineate the signaling networks associated with Tex^int^. Our analysis revealed that several signaling pathways, including SPP1, EGF, LIGHT, CD70, and VEGF, were predominantly upregulated in LUAD patients compared to normal controls (Fig. S4A, B). Additionally, the number of interactions in Tex^int^ were markedly increased in LUAD samples (Fig. 4A). Particularly, the incoming signals including SPP1, CXCL, CD70, LIGHT, and CD137 were exclusively observed in Tex^int^ of LUAD. In outgoing signals, the unique signals from Tex^int^ of LUAD patients were CXCL, LIGHT, CD70, EGF, and GRN (Figs. 4B and S5A). Therefore, we proposed that these signals might be crucial for the functions of Tex^int^ in LUAD (Fig. 4B). Intriguingly, CD137 signaling exhibited the strongest incoming signal to Tex^int^ compared to the other cell types (Figs. 4C and S5A, B). Tex^int^ predominantly received CD137 signals from plasma cells and CD8_01 cells (Fig. 4D). In addition, *TNFRSF9* (encoding CD137) and *TNFSF9* (encoding CD137L; ligand) were highly expressed in Tex^int^ and CD8_01 respectively, suggesting that Tex^int^ mainly receive CD137 signaling from CD8_01 (Fig. 4E). Our findings elucidate 1CM-high proliferative Tex^int^ mainly receive CD137 signaling, which might contribute to terminal T cell exhaustion and poor immunotherapy response [51].

### Proliferative Tex^int^ are enriched in LUAD tumors from non-responders to anti-PD-1 therapy

**Fig. 5:**
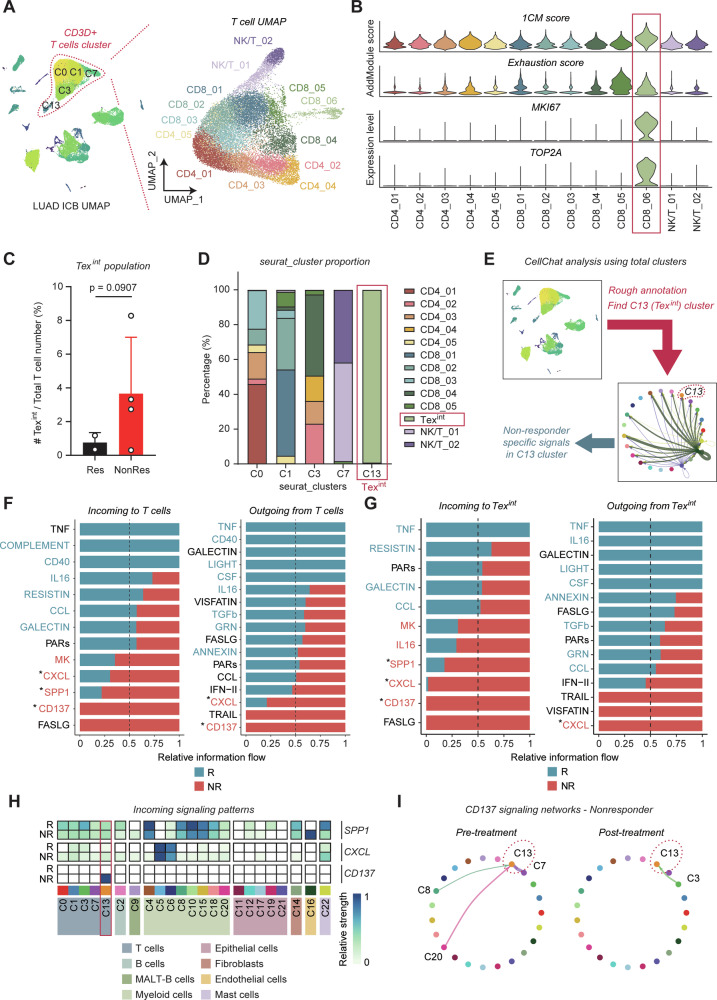
Proliferative Tex^int^ are enriched in LUAD tumors from non-responders to anti-PD1 therapy. **A** Uniform Manifold Approximation and Projection (UMAP) shows total cluster of ICB-treated LUAD patients (left) and re-clustered CD3D+ T cell cluster (C0, C1, C3, C7, C13) (right). **B** Violin plot represents 1CM score, exhaustion score, and expression level of proliferation markers *MKI67*, *TOP2A* in overall CD3D+ T cell. **C** Bar plot shows an increased proportion of Tex^int^ in non-responders to anti-PD-1. *p* value was calculated by unpaired *t-*test with Welch’s correction. **D** Bar plot exhibits distribution of original seurat cluster (C0, C1, C3, C7, C13) in re-clustered T cells. Although C0, C1, C3, and C7 show heterogeneous composition, C13 exhibits dominant proportion of Tex^int^ cluster. **E** Schematic diagram shows workflow of cell-to-cell communication analysis. Total cell clusters are roughly annotated by marker genes, and we investigated unique signaling for C13 cluster, representing Tex^int^ cluster. Bar plot shows the ranking of major incoming or outgoing signaling of overall T cells (**F**) and Tex^int^ (**G**) between responders and non-responders. The rank was based on differences in relative information flow, which was calculated by the total weights in the cellular network. R indicates responders, and NR indicates non-responders. **H** Heatmap shows interaction patterns of incoming signals in responders and non-responders. CD137 signaling was not detected in responder group. R indicates responders, and NR indicates non-responders. **I** Circle plot shows CD137 signaling network in pre and post anti-PD-1 non-responders.

We next analyzed scRNA-seq data from LUAD patients, including responders and non-responders to anti-PD1 treatment. To examine whether 1CM-enriched proliferative Tex^int^ exist in LUAD TIME from non-responders, we re-clustered T cells expressing *CD3D* across clusters C0, C1, C3, C7, and C13. We identified five CD4 T cell clusters (CD4_01-05), six CD8 T cell clusters (CD8_01-06), and two NK/T cell clusters (NK/T_01, 02) (Figs. 5A and S6A). In addition, we roughly annotated other seurat clusters using known markers; B cells (*MS4A1*), MALT-B cells (*MZB1*), Myeloid cells (*FCGR3B*, *CD86*), Epithelial cells (*EPCAM*), Fibroblasts (*TAGLN*), Endothelial cells (*VWF*), Mast cells (*KIT*) (Fig. S6B). Notably, CD8_06 exhibited phenotypic similarities with Tex^int^ (Figs. 5B and S6A, C). In addition, CD8_06 cluster contained 92.4% of Tex^int^ which were negatively enriched with Tpex signature score, meaning that Tex^int^ cells are dominant in CD8_06 cluster (Fig. S6D). Their proportion was increased in anti-PD1 non-responders, implicating a potential link between increased cell numbers of Tex^int^ and poor immunotherapy response (Fig. 5C).

We next performed cell-cell communication analysis to investigate CD137 signaling network in Tex^int^ cells. To mitigate statistical biases arising from small cluster sizes, we utilized original seurat clusters rather than those generated from re-clustering. Given that 99.2% of C13 consisted of Tex^int^ cells, we designated C13 as representative of Tex^int^ cells (Fig. 5D). This annotation enabled us to identify signals specific to non-responder within Tex^int^-enriched C13 cluster (Fig. 5E). Both overall T cells and Tex^int^ cells of non-responders to anti-PD1 exhibited upregulation of three signals (CXCL, SPP1, CD137), which overlapped with five incoming signals to Tex^int^ cells as mentioned in Fig. 4B (Fig. 5F, G). Particularly, CD137 signaling was exclusive to non-responders and exhibited its strongest expression in Tex^int^ cells compared to other cell types, while SPP1 and CXCL signals displayed stronger interactions in different cell types (Figs. 5H and S6E, F). Circle plot depicts that CD137 signaling was uniquely received by Tex^int^ cells in non-responders, both pre and post treatment of anti-PD1, whereas such signaling was absent in responders (Fig. 5I). These results suggest that CD137 signaling might play a key role to promote exhaustion and 1CM-mediated proliferation in Tex^int^ cells, potentially contributing to the persistence of Tex^term^ cells and immune escape in LUAD non-responders to anti-PD1.

### 1CM-high proliferative Tex^int^ are prominently distributed in immunotherapy non-responders of various cancer types

**Fig. 6:**
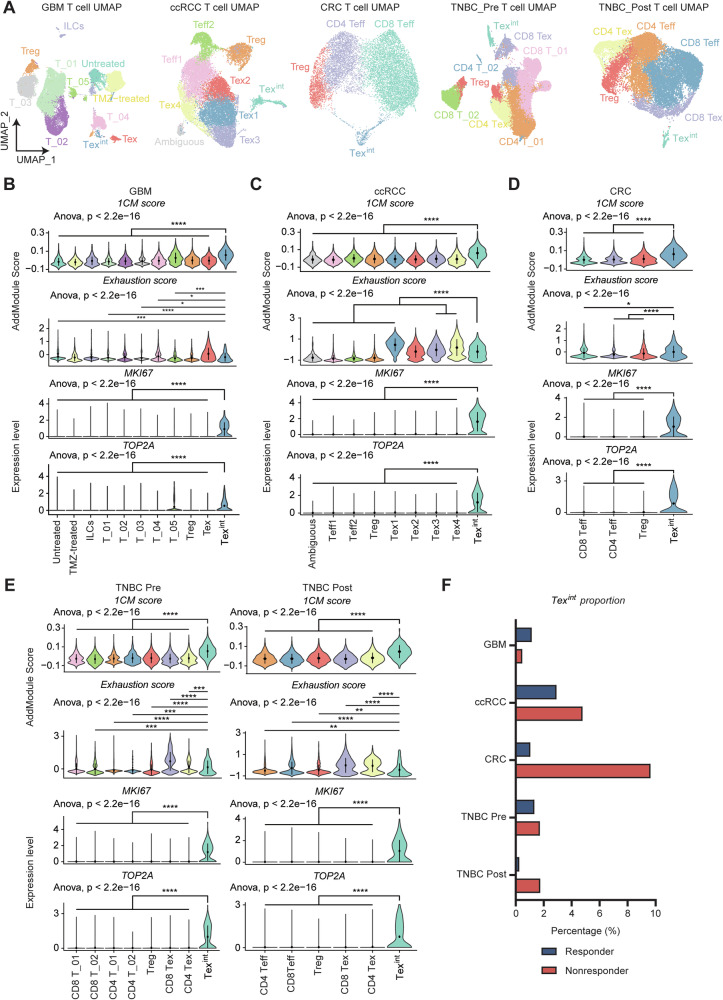
1CM-enriched proliferative Tex^int^ cells are dominant in ICB therapy non-responders of various cancer types. **A** Each Uniform Manifold Approximation and Projection (UMAP) from GBM, ccRCC, CRC, TNBC_Pre, and TNBC_Post shows diverse types of T cells including Tex^int^. The anticancer drugs administered to each cancer patient are as follows (GBM; anti-PD-1, ccRCC; anti-PD-1, CRC; anti-PD-1 and celecoxib, TNBC; Atezolizumab and paclitaxel). Violin plots depict 1CM score, exhaustion score, and expression level of proliferation markers, *MKI67* and *TOP2A*, in T cell subtypes of GBM (**B**), ccRCC (**C**), CRC (**D**), and TNBC_Pre and Post (**E**). Multiple comparison analysis was performed using ANOVA and two-group comparison was performed by Wilcoxon rank-sum test. **F** Bar plot shows proportion of Tex^int^ are almost increased in non-responder group of diverse types of cancer.

We subsequently analyzed several scRNA-seq data from patients treated with immune checkpoint blockade (ICB) across different cancer types to comprehensively extend our findings. We retrieved and utilized public scRNA-seq data of glioblastoma (GBM), clear cell renal cell carcinoma (ccRCC), colorectal cancer (CRC), and triple negative breast cancer (TNBC) patients. Each dataset was processed to isolate *CD3D*+ T cells, followed by dimensionality reduction using Uniform Manifold Approximation and Projection (UMAP) (Fig. 6A). Next, we assessed exhaustion scores and several proliferation markers such as *MKI67*, *TOP2A* to identify Tex^int^ cluster within each dataset (Fig. 6B–E). Also, we investigated *TCF7*, *TOX* expression level and Tpex signature score, and finally confirmed Tex^int^ cluster in all datasets (Fig. S7A–D). Remarkably, the Tex^int^ cluster of all datasets displayed high 1CM scores, indicating that association between Tex^int^ cells and 1CM is observed in a broad manner including patients with LUAD (Fig. 6B–E). Furthermore, the proportion of Tex^int^ cells was higher in non-responders compared to responders in all datasets, except for GBM (Fig. 6F). Interestingly, in the case of TNBC, the proportion of Tex^int^ was indistinguishable between responders and non-responders before treatment. In treatment groups, this proportion significantly decreased in responders, whereas it increased in non-responders, suggesting that increased proportion of Tex^int^ could be associated with poor immunotherapy response (Fig. 6F). However, CD137 expression was not observed or increased in Tex^int^ cells of non-responders across all datasets, suggesting that main signaling, contributing to terminal exhaustion of Tex^int^ cells, could be different according to cancer types. Collectively, our findings suggest that 1CM represents a distinct metabolic feature of Tex^int^ cells in diverse cancer types.

## Discussion

1CM has been emerging as a therapeutic target in various cancer types. Traditionally, numerous studies on 1CM and its role in cancer have primarily focused on cancer cells, typically epithelial cells across different cancer types. However, recent research has demonstrated that the tumor immune microenvironment (TIME) plays a critical role in regulating cancer progression and resistance to anticancer therapies. Therefore, it is crucial to explore the underlying mechanisms of how 1CM modulates the biological functions of TIME to affect the efficacy of anticancer therapies, including immunotherapies [56–58].

Using scRNA-seq data from lung adenocarcinoma (LUAD) patients, we found that 1CM was enriched in T cells within LUAD tissues compared to normal tissues. Furthermore, 1CM, specifically the mitochondrial folate cycle, was significantly enriched in the Tex^int^ cluster, and its proportion was increased in LUAD samples. These Tex^int^ cells were highly proliferative and expressed exhaustion markers such as *PDCD1*, *HAVCR2*, and *LAG3*. Thus, we hypothesize that 1CM, particularly the mitochondrial folate cycle, is crucial for Tex^int^ proliferation. Recent reports also indicated that *SHMT2* and *MTHFD2*, which are linked to the mitochondrial folate cycle, contribute to T cell exhaustion. Mitochondrial dysfunction, induced by glycolytic reprogramming, promotes the transition of Tex^int^ into terminally exhausted T cells (Tex^term^) [52, 53, 59].

Although we focused on the mitochondrial folate cycle, other downstream pathways in 1CM, such as the glutathione (GSH) synthesis pathway, thymidine synthesis, and the methionine cycle, might also play key roles in T cell exhaustion and the transition of Tex^int^ to Tex^term^. Hypoxia-induced mitochondrial stress has been well-known to promote T cell exhaustion [60]. Activated T cells also exhibit increased glucose utilization and ATP generation by the mitochondrial electron transport chain for their clonal expansion, resulting in an increase of reactive oxygen species (ROS) levels [61–64]. Therefore, both Tex and activated T cells need antioxidants, such as GSH, to protect themselves from ROS damage [61]. Hence, 1CM-induced GSH synthesis may protect Tex^int^ from hypoxic conditions during their exhaustion process. Purine/thymidine synthesis by 1CM also supports rapid T cell expansion by providing DNA building blocks [65]. Furthermore, increased extracellular acidosis restricts 1CM, methionine metabolism, and T cell exhaustion, helping maintain a T cell stem-like memory state and anti-tumor efficacy [66]. Altogether, our findings and previous studies highlight the importance of the 1CM-proliferation axis in Tex^int^. However, further bioinformatic analysis is needed to fully understand the multifunctional role of 1CM in TIME.

Our cell-cell communication analysis revealed that 1CM-enriched Tex^int^ cells were significantly modulated by the incoming CD137 signaling pathway. CD137 is well known as a co-stimulatory molecule for T cell activation, supporting TCR signaling and B7 family-CD28 co-stimulation, which eventually promotes the clonal expansion of activated T cells [40, 42]. However, TCR-independent CD137 stimulation also promotes Tex cell expansion and differentiation, especially in cells that undergo exhaustion program [51, 67]. Therefore, CD137 signaling is crucial for proliferation in both Tex and activated T cells.

Although we did not clarify the exact mechanism by which CD137 signaling increases in non-responders to immunotherapy and the correlation between CD137 signaling and 1CM, we demonstrated that CD137-high Tex^int^ cells predominantly exhibit 1CM metabolism and their population are significantly increased in non-responders. However, in various studies, it remains controversial whether the function of Tex^int^ is helpful to immunotherapy response or not [16, 18, 34–39]. We think the immune microenvironment surrounding Tex^int^ determines the fate of Tex^int^. Namely, whether antigen presentation by DCs is active in TDLNs or whether signals promoting T cell exhaustion, such as CD137, are prevalent could determine the maintenance of cytotoxicity or rapid transitions into Tex^term^ in Tex^int^ [16, 18, 34–39]. This finding can be leveraged as a new strategy to regulate the proliferation of Tex^int^ cells and prevent the expansion of Tex^term^ cells in immunotherapy non-responders. Further analysis is necessary to verify whether inhibition of 1CM can reduce the CD137 signaling-mediated expansion of Tex^int^ cells. Additionally, we found that Tex^int^ also received CD137 signaling from plasma cells in LUAD patients (Fig. 4F). However, the relationship between CD137 signaling and plasma cells has not been well studied. Since we focused on interactions among T cells, we did not analyze the functional roles of plasma cells in T cell exhaustion. Thus, further studies on the interactions between plasma cells and CD137 signaling in TIME could yield valuable insights into T cell exhaustion.

scRNA-seq data from anti-PD1-treated patients in LUAD supported our findings and their clinical implications. We found that the proportion of Tex^int^ cells increased in TIME of anti-PD1-resistant LUAD patients. These Tex^int^ cells exhibited the highest 1CM activities and incoming CD137 signaling compared to other T cells. Thus, we deduced that the CD137 signaling and 1CM enrichment in Tex^int^ might contribute to immunotherapy resistance in LUAD. Additionally, it has been reported that CD137 signaling mediates negative immunoregulation, and patients with high CD137+ CD8+ T cells exhibit poorer survival in lung cancer [68]. We also investigated whether this CD137-1CM-proliferation axis in Tex^int^ can be extended to various cancer types.

**Fig. 7:**
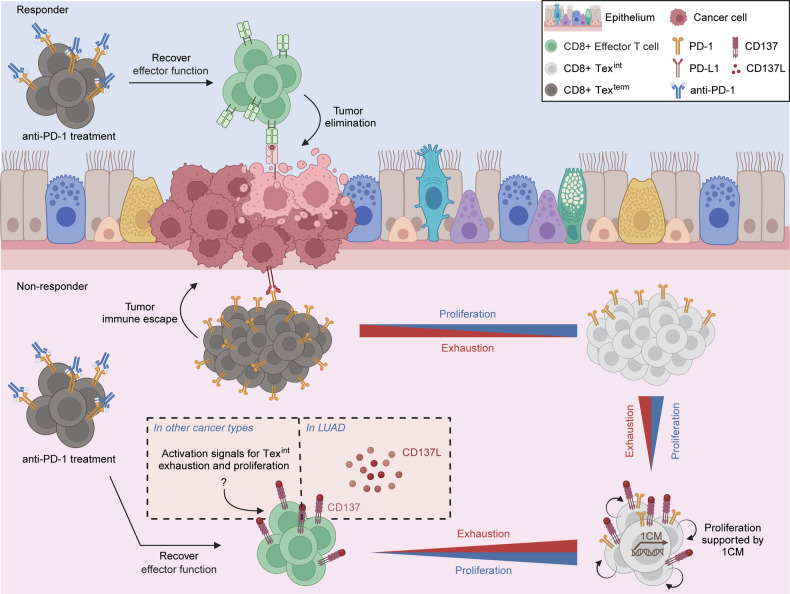
Schematic diagram for CD137-1CM-proliferation axis in LUAD non-responders to ICB therapy. In responders, CD8+ exhausted T cells can recover their cytotoxicity against cancer cells by ICB treatment such as anti-PD-1. In non-responders, CD137-induced differentiation and proliferation of Tex^int^ might diminish effects of ICB treatment. In addition, this CD137-induced Tex^int^ proliferation is mainly supported by 1CM, which we call CD137-1CM-proliferation axis. CD137-1CM-proliferation axis is a distinct feature for Tex^int^ of LUAD non-responders to ICB treatment and can be therapeutic target for anti-PD1 resistant LUAD patient.

However, CD137 signaling was not detected or was even increased in responders in other cancer types. These heterogeneous results, regarding CD137 signaling, likely result from its dual roles to control both T cell activation and exhaustion. Also, these results suggest that the main signaling pathway, which contributes to activate Tex^int^ proliferation and terminal exhaustion, is different according to the cancer type. Although CD137 signaling network in Tex^int^ of other cancer types was not detected unlike the patients with LUAD, it was highly conservative in various cancer types that 1CM was upregulated in proliferative Tex^int^, which were enriched in ICB-treatment non-responders. Thus, we propose that 1CM may be a distinct metabolic signature of Tex^int^ in various cancer types, which is critical for maintaining their proliferation abilities and contributes to poor immunotherapy responses. In order to improve the immunotherapy resistance, we need to clarify which main signal might contribute to enhancement of Tex^int^ proliferation with 1CM in each cancer type (Fig. 7).

Despite numerous efforts, effective treatments to enhance immunotherapy response have not yet been standardized. This is likely due to a lack of understanding about the molecular alterations in generation of exhausted T cells. Here, we propose a new potential strategy of controlling the Tex^int^ proliferation to prevent the re-expansion of Tex^term^. Targeting 1CM, a distinct signature in Tex^int^, could be a novel therapeutic target for immunotherapy non-responders. Additionally, in the case of LUAD, targeting both 1CM and CD137 signaling could provide a more effective approach for immunotherapy non-responders. Further bioinformatic analysis would be required to identify the detailed mechanisms underlying the enhancement of the 1CM-enriched molecular feature to expand Tex^int^ cell population across various cancer types.

## Materials and methods

### Bioinformatic analysis of gene expression and survival time in LUAD patients from public datasets

We utilized two LUAD datasets from the GSE10072 and GSE19804. Significant different one carbon metabolic process between patients with LUAD and normal subjects was confirmed based on DEGs with a log2FC value exceeding 0.4 in Database for Annotation, Visualization and Integrated Discovery (DAVID) (website: https://david.ncifcrf.gov/). factoextra (version 1.0.7) FactoMineR (version 2.11) in R software (version 4.3.3) were used for Principal component analysis of one carbon metabolic process based on GO biological process from MsigDB database. Protein expression associated with one carbon metabolic process was acquired from University of Alabama at Birmingham Cancer data analysis portal (UALCAN; website: https://ualcan.path.uab.edu/). Survival plots associated with one carbon metabolic process genes or pathways were estimated in OncoLnc (website: http://www.oncolnc.org/) and cSurvival (website: https://tau.cmmt.ubc.ca/cSurvival/). All the data related to expression survival were visualized using “GraphPad Prism6”.

### Processing of single cell RNA-seq RAW data

Raw count matrix was downloaded and processed into proper format; cell ids as columns and gene symbols (features) as rows. Processed count matrix was used as input in “CreateSeuratObject” function in “Seurat” (version 5.0.3) in R (version 4.3.1) [69]. If the authors of data source previously uploaded processed Seurat object, we downloaded and used its dimension reduction and cell type annotation information.

### Quality control and standard integration workflow of data

Cells with <200 of nCount_RNA (total number of molecules detected per cell), large amount of nCount_RNA, and >20% of mitochondrial genes were eliminated, regarded as dead cell and doublet, respectively. In addition, we used “DoubletFinder” (version 2.0.4) package to identify and remove doublets. If the original data had already been filtered by the authors, we used that samples without additional quality control procedures.

Then, we performed normalization using “NormalizeData” function in default settings and top 2000 highly variable genes were figured out using “FindVariableFeatures” with selection method “vst”. Using these 2000 variable genes, each dataset was scaled via “ScaleData” function. The anchors for integration were detected using function “FindIntegrationAnchors”. Subsequently, the datasets were combined into single Seurat object using “IntegrateData”.

### Clustering and dimension reduction

To cluster and visualize data, we first performed principal component analysis (PCA) using “RunPCA“ function. Then, the dataset was clustered by “FindNeighbors” and “FindClusters”. Finally, the data is visualized by t-distributed Stochastic Neighbor Embedding (t-SNE) and Uniform Manifold Approximation and Projection (UMAP), well known method for dimension reduction, using “RunTSNE” and “RunUMAP”.

### Cluster annotation

To identify cell type of each cluster, we used “FindAllMarkers” function and identified only positive marker genes with >0.25 of log fold change (logFC). One cluster which has multiple mitochondrial genes in the top ranks of its marker genes were removed, regarded as dead cell clusters.

Then, using well known reference marker genes for lung cell annotation, we compared them with our marker genes list and checked their expression in our dataset using “VlnPlot” and “DotPlot”.

### Data imputation

To handle a lot of missing values in single cell datasets, we performed zero-preserving imputation by low rank approximation using “RunALRA” function in the R package “SeuratWrappers” (version 0.3.1) with default settings. We used imputated expression values in analyzing exhaustion markers of T cells from GSE123904 LUAD dataset in Figs. 3 and S2, 3.

### Single sample gene set enrichment analysis (ssGSEA)

The R package “escape” (version 1.99.1) [70] and “AddModuleScore” function in Seurat package were used to run single sample gene set enrichment analysis (ssGSEA). Gene sets used for ssGSEA were derived from Hallmark gene sets (“H”), Gene Ontology Biological Process (“C5”, “GO:BP”), Kyoto Encyclopedia of Genes and Genomes (“C2”, “KEGG”) based on MSigDB database using “getGeneSets” function in escape.

Calculated enrichment scores were stored in new ident of Seurat object, mapped with each cell. These scores were visualized into violin plot, dot plot, and heatmap by “VlnPlot”, “DotPlot”, and “heatmapEnrichment” functions.

### Metabolism enrichment analysis

We performed ssGSEA for scoring metabolism activity using diverse gene sets from GOBP, KEGG, and HALLMARK. Specific information of gene sets used for ssGSEA is available in Supplementary Table 1. “AddModuleScore” function was used to calculate enrichment score. Calculated score was visualized in dot plot using “DotPlot” function.

We also performed ssGSEA for all metabolism-related KEGG pathways to identify metabolism preferences in each cell types. “runEscape” function was used to calculate enrichment score. Calculated enrichment score was visualized in heatmap using “heatmapEnrichment” function.

### 1CM score, exhaustion score, and Tpex score calculation

We used “AddModuleScore” to perform ssGSEA and scored 1CM activities, exhaustion level, and expression level of Tpex signature genes. GOBP gene set “GOBP_ONE_CARBON_METABOLIC_PROCESS” was used to score 1CM activities, *PDCD1*, *HAVCR2* (encoding TIM-3), and *LAG3* were used to score exhaustion level, and *TCF7* (encoding TCF-1), *CXCR5*, *SLAMF6* (encoding Ly108), *NT5E* (encoding CD73), *ID3* were used to score Tpex signature.

### Pseudotime analysis based on single-cell trajectory analysis

The R package “slingshot” (version 2.10.0) was used to perform single-cell trajectory analysis. SingleCellExperiment object was generated by “as.SingleCellExperiment” function [71]. We next load dimension t-SNE dimension reduction and annotated cluster information in this object. Pseudotime was calculated by “slingshot” function with setting CD4_02 (Naïve T cell) cluster as root using “start_clus = ‘CD4_02’” arguments.

Four lineages were detected and their calculated pseudotimes were visualized as color gradient plotted on their t-SNE. The cells included in lineage 1, which contains Tex^int^ and Tex^term^ clusters, were extracted from original Seurat object by “subset” function. Calculated pseudotime for lineage 1 cells was imported into extracted Seurat object, and correlation between pseudotime and gene expression level was visualized as scatter plot using “FeatureScatter” function.

### Display of cell type proportion

We displayed cell type proportion as bar plot with comparison between two groups: LUAD vs. Normal or Responders vs. Non-responders. In each group, cell type proportion was obtained from total number of target cell type divided by total number of T cells.

### Cell-cell communication analysis

For cell-cell communication analysis, LUAD scRNA-seq data and ICB-treated LUAD scRNA-seq data were examined using the R package “CellChat” (version 1.6.1) [72]. To identify differentially expressed signals between two groups (LUAD vs. Normal, Responders vs. Non-responders), we created and processed each Cellchat object separately, and merged using “mergeCellChat” function. Number of outgoing/incoming interactions from/to Tex^int^ were calculated and visualized by “netVisual_circle” function. Using “rankNet” function, we next identified information flow which were differentially expressed in overall T cells and Tex^int^ of two groups. We next identified interaction patterns of outgoing/incoming signals among the cell types using “netAnalysis_signalingRole_heatmap” function. In addition, these interaction patterns of certain signaling network among the cell types were also visualized into circle plot by “netVisual_aggregate” function.

## Supplementary information

Supplementary Figure

Supplementary Table

## Data Availability

We analyzed two bulk microarray data from tumor tissues and adjacent normal tissues of LUAD patients. The datasets analyzed during the current study are available in the Gene Expression Omnibus (GEO) database, GSE10072 and GSE19804, respectively [73, 74]. We analyzed a scRNA-seq dataset, consisting of only primary tumor samples from treatment-naive LUAD patients (*n* = 7) and normal lung tissue biopsies (*n* = 4). The datasets analyzed during the current study are available in the Gene Expression Omnibus (GEO) database, GSE123904 [75]. We also analyzed five scRNA-seq data of patients from different cancer types received immunotherapy; LUAD, GBM, ccRCC, CRC, and TNBC. The datasets analyzed during the current study are available in the Gene Expression Omnibus (GEO) database and European Genome-Phenome Archive (EGA), GSE207422, GSE235676, EGAD00001008166, GSE205506, and GSE169246, respectively [76–80].
