# Supplementary material for: One-carbon metabolism is distinct metabolic signature for proliferative intermediate exhausted T cells of ICB-resistant cancer patients
Source: Cell Death Discov. 2025 Feb 14;11:60. doi: 10.1038/s41420-025-02332-z (PMC11829039; doi:10.1038/s41420-025-02332-z)
Supplement: Supplementary file 1 — Supplementary Figure [file 41420_2025_2332_MOESM1_ESM.docx]

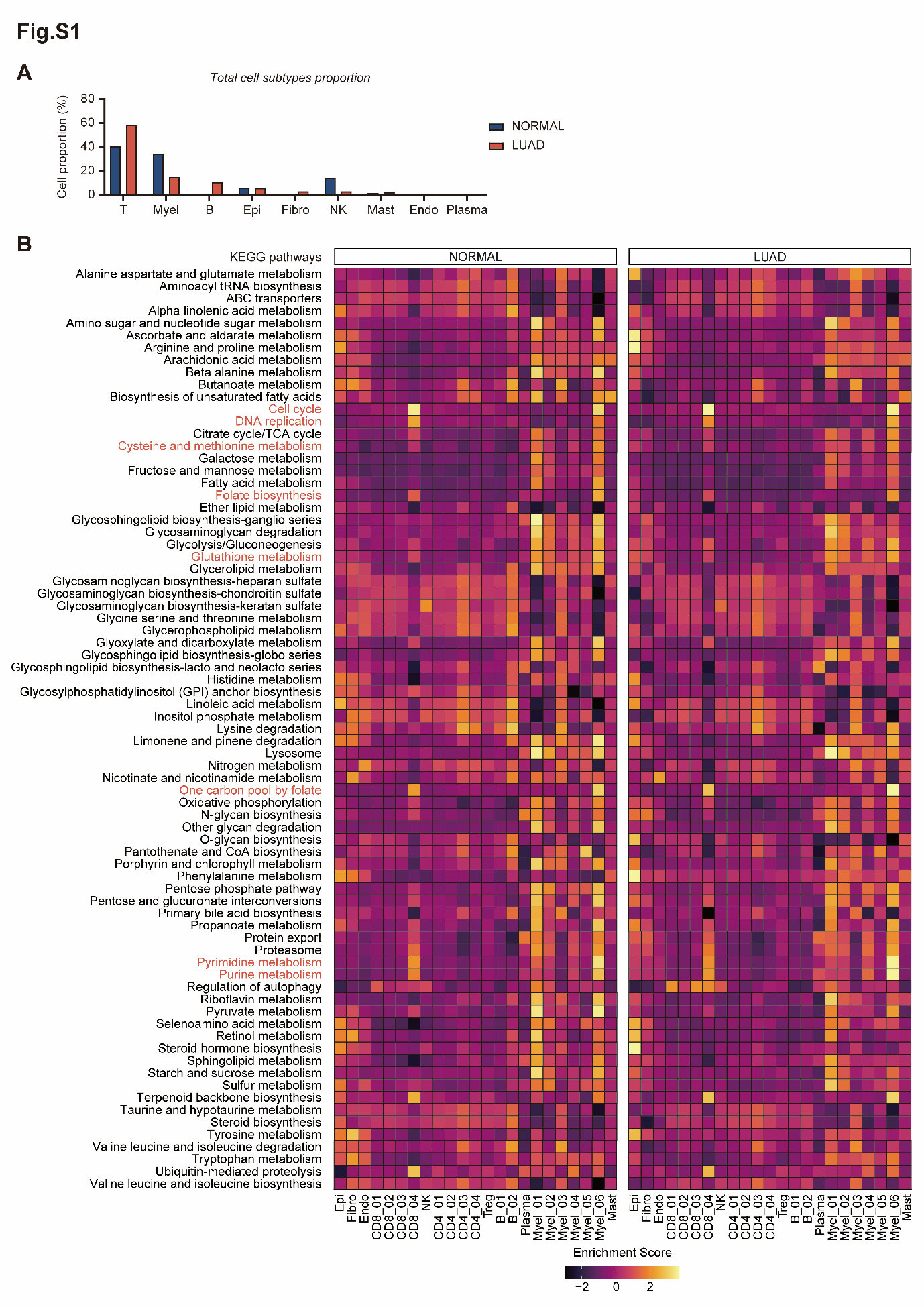


**Supplementary Fig. 1 Heterogeneity in cell types and their expression of metabolic pathways in LUAD patients A** Bar plot shows T cells have the largest portion in TME of LUAD patients. **B** Heatmap depicts difference in enrichment score for all KEGG pathways related to metabolism. 1CM-related pathways are highlighted as red color.


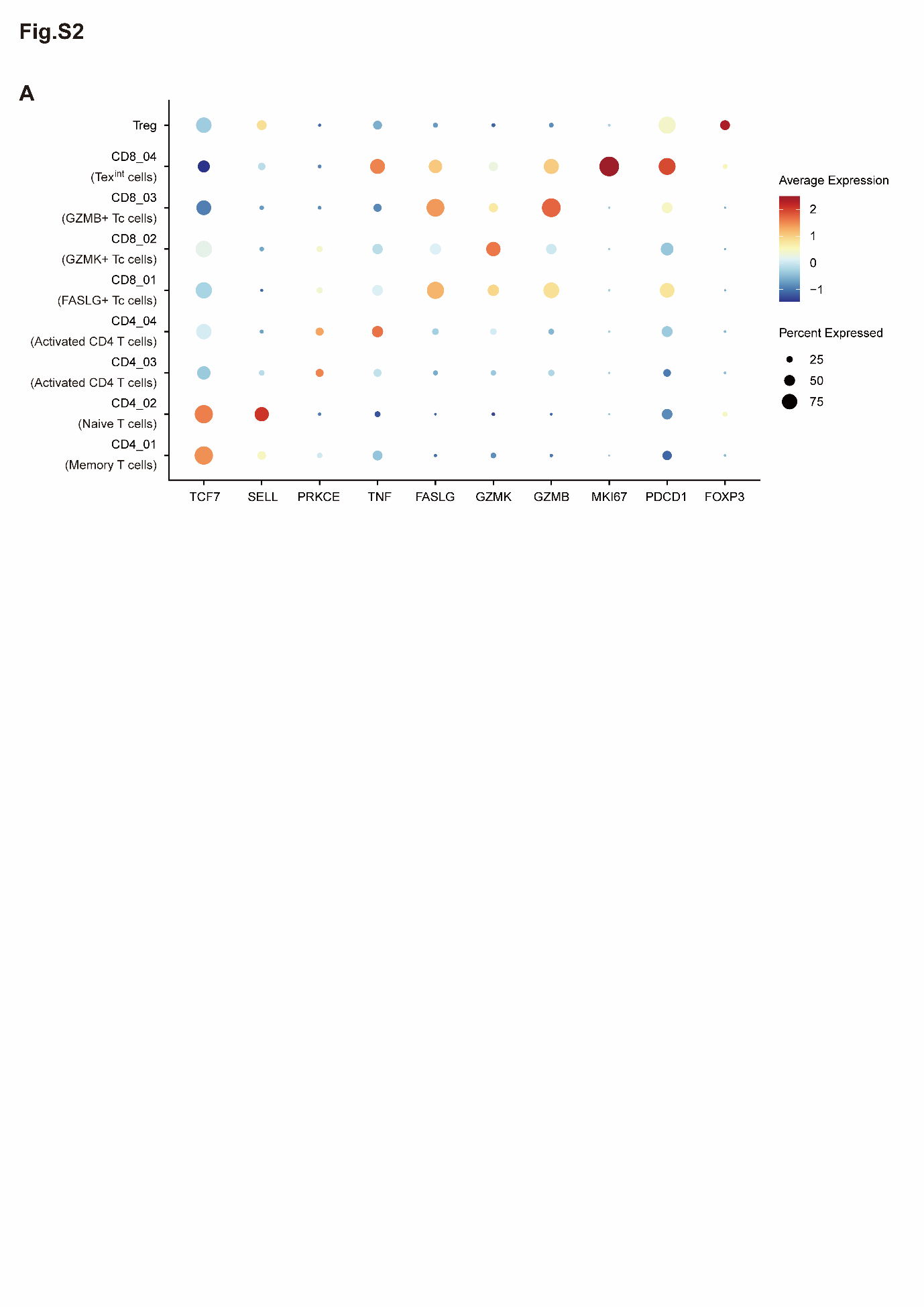


**Supplementary Fig. 2 Identification of T cell subtypes in LUAD patients by expression of marker genes A** Dot plot displays average expression of marker genes for T cell subtypes. CD8_04 cells have the highest expression of both proliferation marker (*MKI67*) and exhaustion marker (*PDCD1*), suggesting the possibility of Tex^int^.


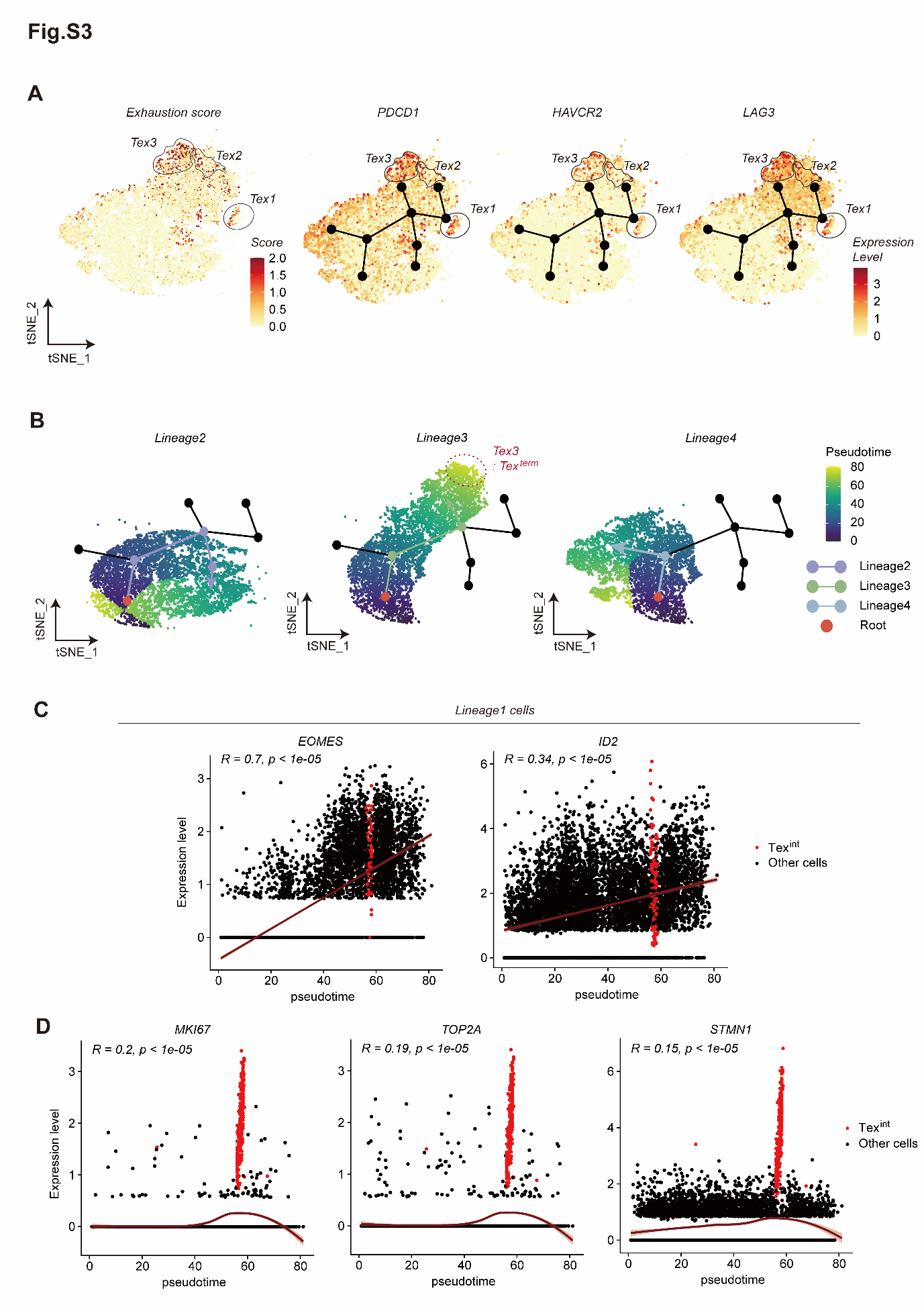


**Supplementary Fig. 3 Pseudotime analysis for T cell lineages identified Tex^int^ cell type, highly expressing both exhaustion markers and proliferation markers A** Tex1, Tex2, and Tex3 clusters are named in feature plot of overall T cell clusters based on their high exhaustion score and original cluster (CD8_04, CD8_03, and CD8_01, respectively). Exhaustion score was calculated by AddModuleScore function in Seurat package with three exhaustion markers, *PDCD1*, *HAVCR2*, *LAG3*. Feature plot displays expression level of each exhaustion marker (*PDCD1*, *HAVCR2*, *LAG3*). **B** t-SNE plot shows lineage information and pseudotime distribution of lineage 2, 3, and 4. **C, D** Correlation plots depict correlation of exhaustion markers (**C**) and proliferation markers (**D**) with pseudotime in lineage 1 cells. Statistical significance was calculated by two-sided t-test.


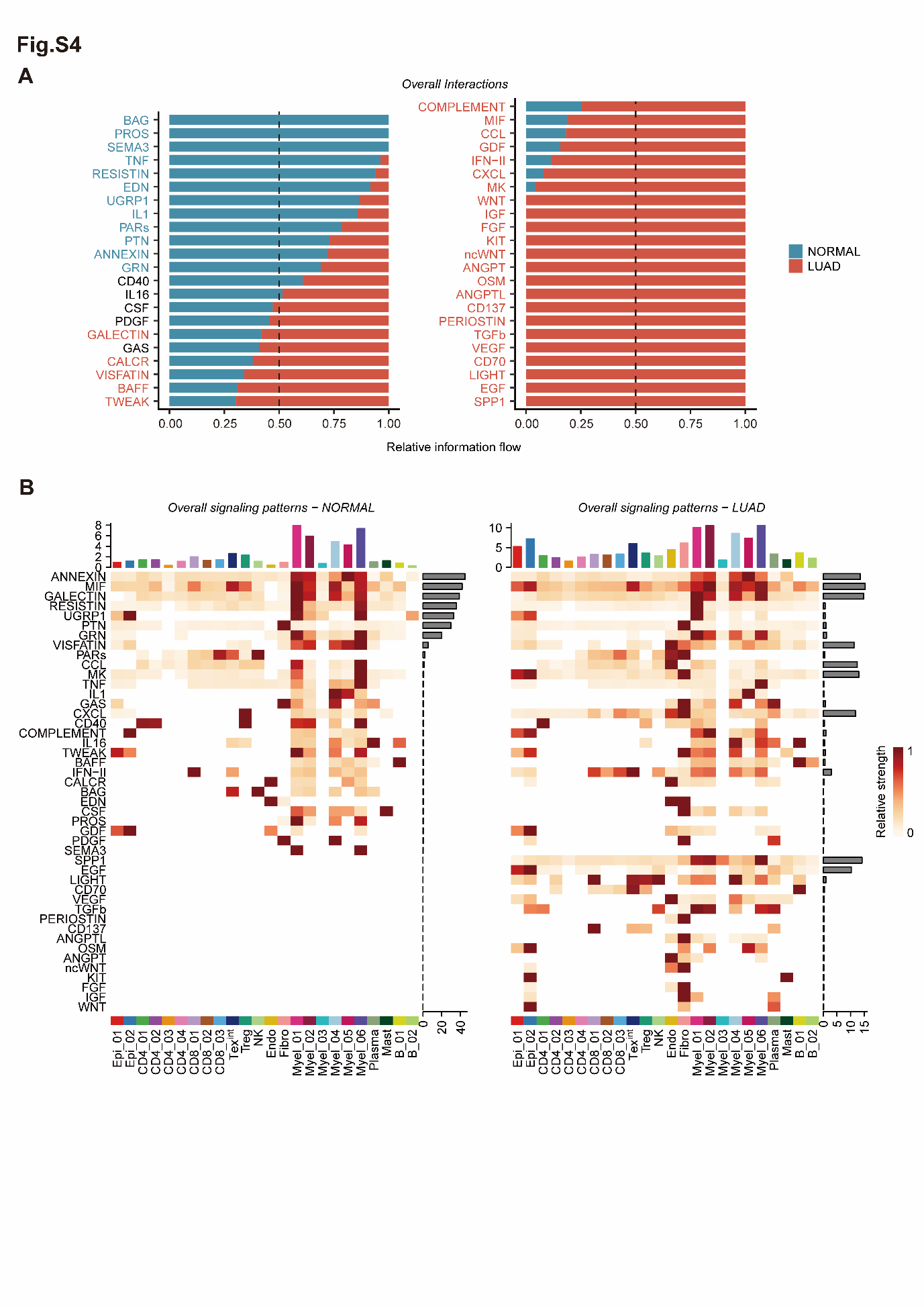


**Supplementary Fig. 4 Different signals were observed between LUAD and normal samples in cell-to-cell communication analysis A** Bar plots show the rank of major signal interactions, including outgoing and incoming signals, for total cell types between LUAD and normal samples. The rank was based on differences in relative information flow, which was calculated by the total weights in the cellular network. **B** Heatmaps depict overall interaction patterns of outgoing signals and incoming signals in LUAD (left) and normal samples (right).


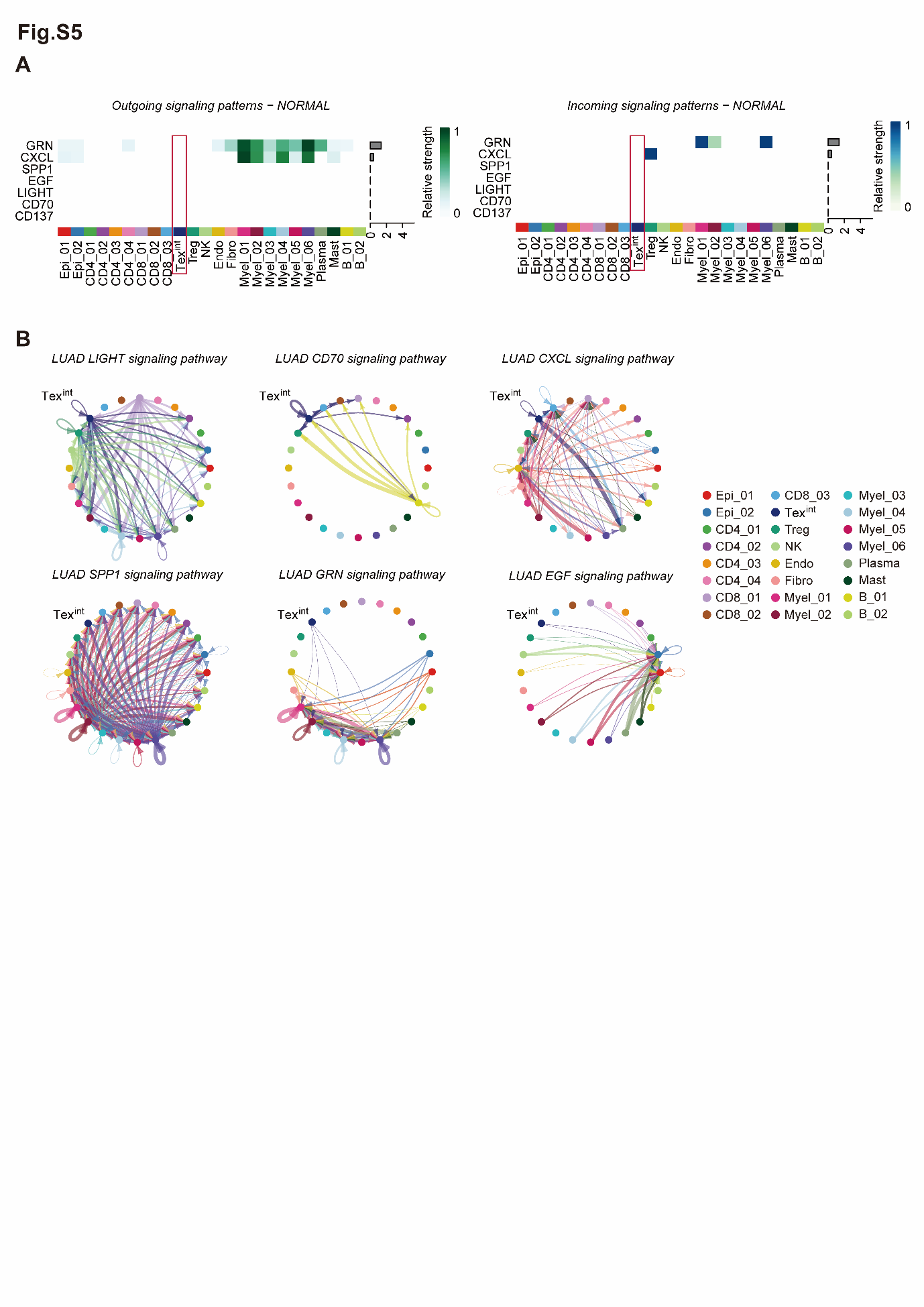


**Supplementary Fig. 5 Signals which are upregulated in LUAD Tex^int^ than normal Tex^int^ were identified and visualized into heatmap and circle plot A** Heatmaps show interaction patterns of outgoing signals (left) and incoming signals (right) in normal samples. Tex^int^ clusters were marked as red box. **B** Circle plots display signaling network and strength of LIGHT, CD70, CXCL, SPP1, GRN, and EGF signals in LUAD.


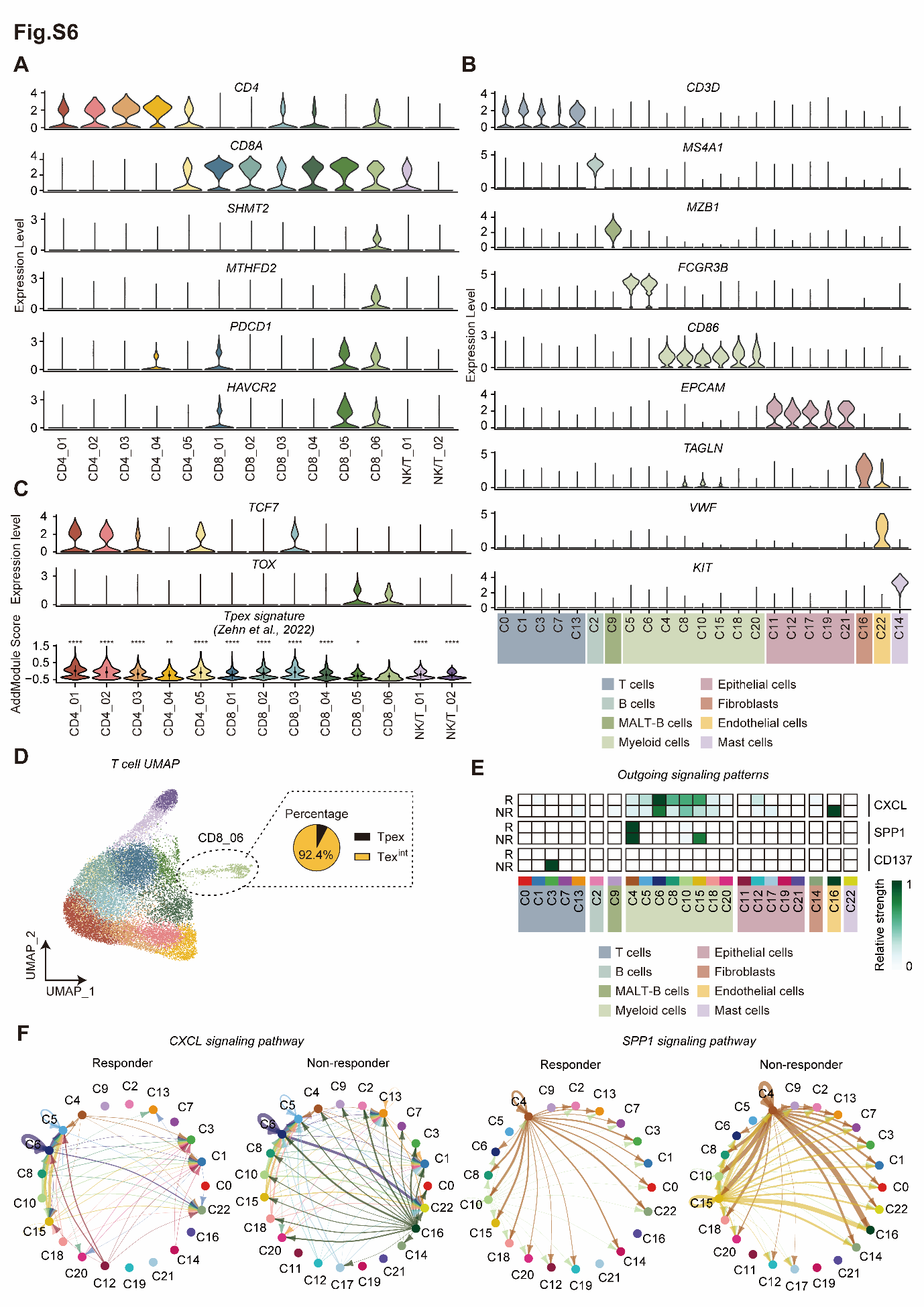


**Supplementary Fig. 6 1CM was highly expressed in Tex^int^ which are enriched in anti-PD-1 non-responders of LUAD A** Violin plots display average expression of T cell receptor molecule (*CD4*, *CD8A*), 1CM-related genes (*SHMT2*, *MTHFD2*), and exhaustion markers (*PDCD1*, *HAVCR2*). **B** Violin plots show average expression of marker genes which were used to roughly annotate total cell clusters. **C** Violin plots depict expression level of canonical Tpex marker *TCF7*, terminal exhaustion and differentiation marker *TOX*, and Tpex signature score (*TCF7*, *CXCR5*, *SLAMF6*, *NT5E*, *ID3*). CD8_06 shows Tex^int^ phenotype (high *TOX* expression, low *TCF7* expression and Tpex signature score). Statistical significance was calculated by Wilcoxon rank-sum test. **D** Pie chart shows the population of Tpex and Tex^int^ in CD8_06 cluster. CD8_06 cluster are constituted with 92.4% of Tex^int^, suggesting that Tex^int^ are dominant in CD8_06 cluster. **E** Heatmap shows interaction patterns of outgoing signals in responders and non-responders. R indicates responders, and NR indicates non-responders. **F** Circle plots depict different signaling network and strength of CXCL, SPP1 signaling between responders and non-responders of LUAD.


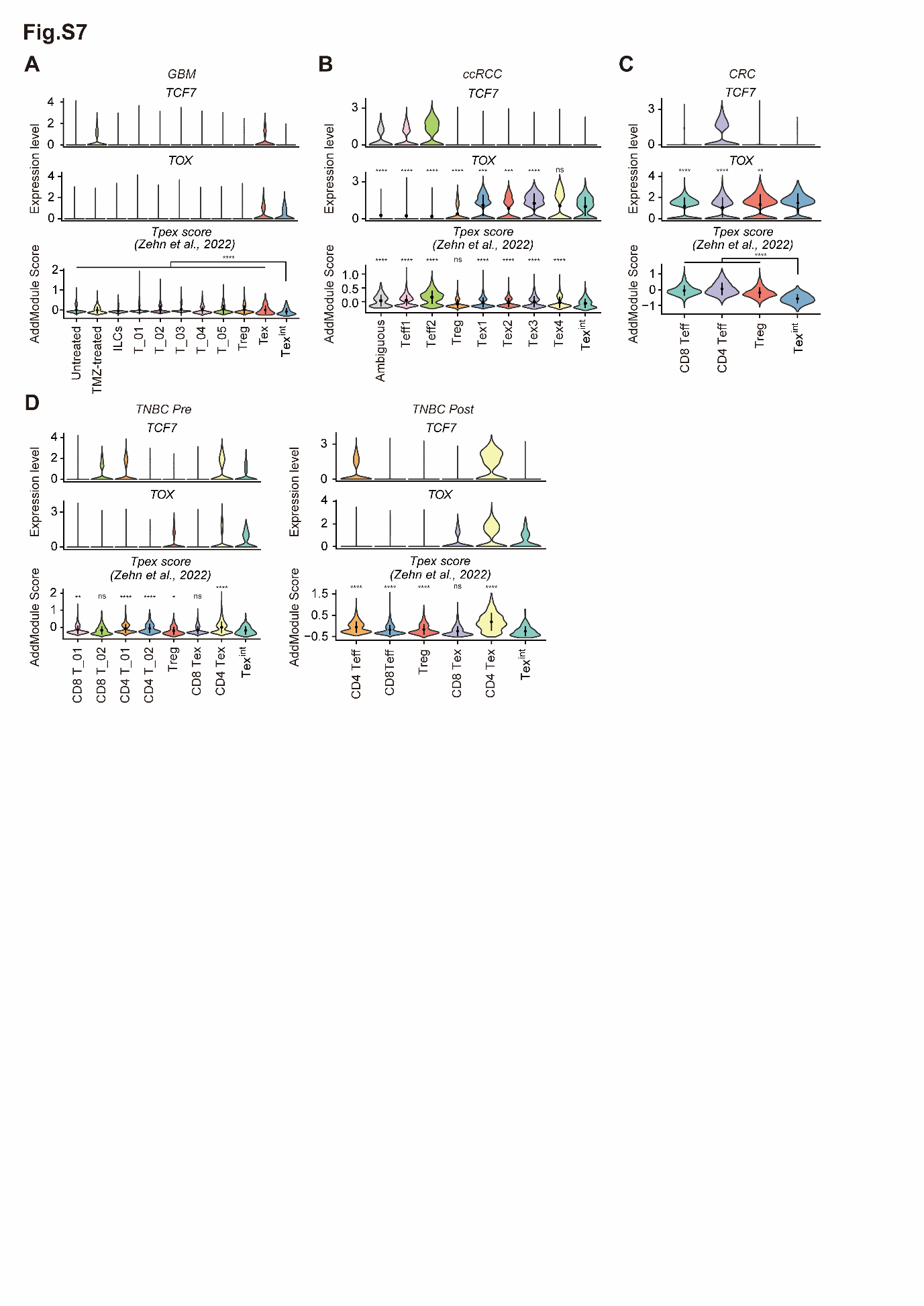


**Supplementary Fig. 7 Identification of Tex^int^ using Tpex, Tex^int^ marker genes and Tpex score in multiple cancer types A-D** Violin plots show expression level of canonical Tpex marker *TCF7*, terminal exhaustion and differentiation marker *TOX*, and Tpex signature score (*TCF7*, *CXCR5*, *SLAMF6*, *NT5E*, *ID3*) among the T cells from GBM (**A**), ccRCC (**B**), CRC (**C**), TNBC_Pre and TNBC_Post patients (**D**). Statistical significance was calculated by Wilcoxon rank-sum test.
